# The clinical observation of none-promyelocytic AML patients inducted with idarubicin or daunorubicin included standard regimens: a tertiary care center experience

**DOI:** 10.1186/s40360-025-00839-w

**Published:** 2025-01-20

**Authors:** Jianhui Xu, Chaoyang Song, Yanjie He, Rui Huang, Sanfang Tu

**Affiliations:** 1 Department of Hematology, Zhujiang Hospital of Southern Medical University, No. 253, Gongye Road, Haizhu District, Guangzhou, 510280 China; 2 Guangdong Engineering Research Center of Precision Immune Cell Therapy Technology, Zhujiang Hospital, No. 253, Gongye Road, Guangzhou, Haizhu District 510282 China

**Keywords:** Acute myeloid leukemia, Idarubicin, Stem cell transplantation, MLL rearrangement, DNMT3A mutation, TET2 mutation

## Abstract

**Background:**

Few Chinese study compared the impacts of idarubicin and daunorubicin based “3+7” intensive chemotherapies on early and long-term outcomes of AML patients through exploring their real-world data.

**Patients and methods:**

Our none promyelocytic AML patients inducted with “3+7” regimens were studied to find out the factors relating with induction response and long term survival.

**Results:**

Idarubicin induction was related with less chemotherapy refractory rate comparing with daunorubicin induction (10% vs 25%, *P* = 0.02). But cytogenetic molecular risk classification was the only independent factor relating with achieving CR after initial induction or chemotherapy refractory (*P* = 0.000 and 0.036). Both to overall survival (OS) and progress free survival (PFS), having transplantation and chemotherapy refractory were independent factors related, MLL rearrangement and DNA methylating related genes’ mutations as well. CR at time of transplantation and MLL rearrangement were independent factors relating both with OS after transplantation and relapse free survival after transplantation.

**Conclusion:**

Traditional “3+7” chemotherapy regimen with idarubicin plays better in CR induction than that with daunorubicin. But the patient’s long-term survival related with clinical practice aspects, like having stem cell transplantation, as well as genetic alterations equally, like MLL rearrangement and DNA methylating related genes’ mutations.

## Introduction

Acute myeloid leukemia (AML) is a malignant clonal disease of hematopoietic cell hierarchy that originated from normal primitive cells rather committed progenitor cells [9]. The pivotal efficient induction regimens are still standard “3+7” chemotherapy which consists of 7 days of continuous cytarabine with 3 days of anthracycline [15]. Daunorubicin (DNR) and idarubicin (IDR) are the most typically adopted anthracyclines except for regimens to those unfit for intensive chemotherapy. Idarubicin is less susceptible to cellular efflux by multidrug resistance proteins [7], and has the virtue of increased duration of drug exposure compared with daunorubicin [31]. Earlier randomized trials did show the superior response of regimen including idarubicin comparing with regimen including standard dose of daunorubicin [4, 6]. But some larger sample trials didn’t verify the different efficiency between daunorubicin and idarubicin [23, 28]. And although the attainment of morphologic complete remission is deemed as a early surrogate of long-term survival, the association between CR and OS is still argued in many AML trials especially in elder patients [8]. The clinical outcomes of AML related with general aspects of patients like age and gender [5, 16], clinical parameters at onset like white blood cell counts, FAB types and cytogenetic risk classifications [12, 14], and treatment aspects like induction chemotherapy regimens, treatment response of complete remission and hemotopoietic stem cell transplantation et al [3, 33].

In china, a few retrospective clinical studies compared induction response of idarubicin and daunorubicin in limited samples. And the idarubicin based regimen showed superior response comparing with daunorubicin based regimen whatever evaluated after one or two cycle of induction [21]. Another Chinese retrospective observation on single arm of idarubicin included regimen reported the 5 years survival of 49.18% and cytogenetic risk or complete remission after one course of induction as independent factors influencing the survival [30]. But few studies in China compared the difference of long-term outcomes of patients who received idarubicin or daunorubicin based regimen. So, we made this retrospective observation of our patients who were inducted with either of the two anthracyclines to explore the different regimens’ impact on the induction response and many other clinical factors’ influences on these patients’ long-term outcomes.

## Material and methods

### Patients

**Fig. 1:**
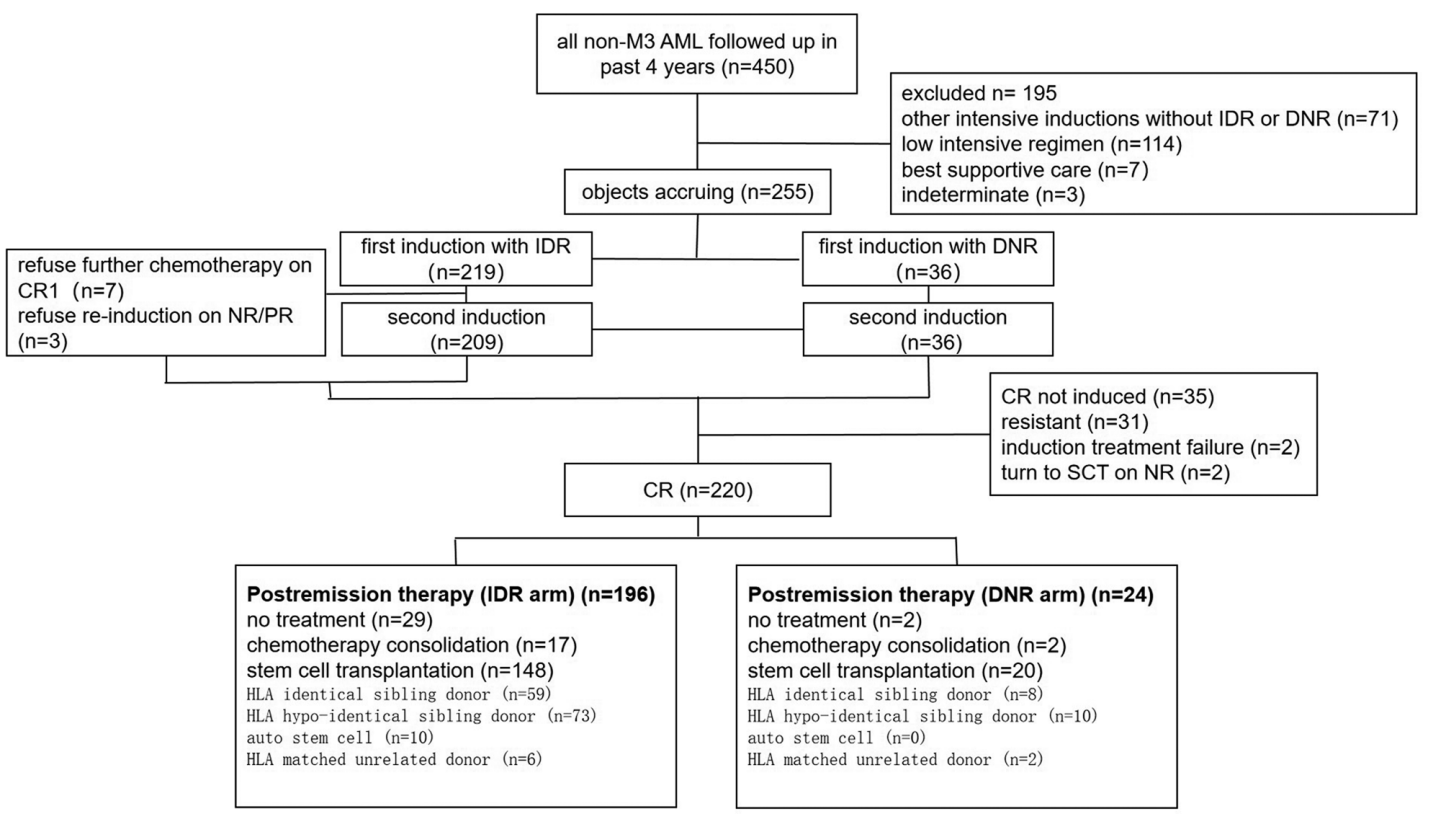
Patients flow diagram, *CR* complete remission, *NR* no remission, *PR* partial remission, *IDR* idarubicin, *DNR* daunorubicin

The observation study was approved by the ethical committee of Zhujiang hospital of southern medical university. All AML patients of non-promyelocytic leukemia who were followed up in our center during past 4 years were reviewed. AML diagnosis was according to the patients’ bone marrow morphological test, karyocyte phenotype test by flowcytometry, karyotype test for bone marrow cells, and new generation sequencing test of AML related prognostic gene panel. The patients initially induced by IDR or DNR based “3+7” regimens were selected. We excluded AML patients with other intensive chemotherapy such as mitoxantron based “3+7”regimen or addition of a third agent like etoposide to the IDR or DNR based “3+7” induction regimen, as well as patients treated by low intensive chemotherapy such as azacytidine, decitabine with or without venetoclax. The inclusion and exclusion criterions were also shown in Fig. 1 of patient flow diagram. The patients were recognized as de novo AML or secondary AML according to history of malignant diseases like nasopharyngeal carcinoma, former myelodysplastic anemia or myeloproliferative neoplasms of essential thrombocythemia, chronic myelogenous leukemia et al. The patients’ induction chemotherapies were underwent from Jun. 2014 to Feb. 2022.

### Inrollment and treatments

Those selected patients according to “3+7” induction chemotherapy whaever in our center or other care center were retrospectively investigated. The first induction treatment should consist of continuous infusion of 100 mg/m^2^ cytarabine each day for 7 days and bolus intravenous injection of 8–12 mg/m^2^ IDR or 45–60 mg/m^2^ DNR daily for 3 days. Patients were divided into IDR and DNR group. Those achieving partial remission after first induction had a repetitive induction regimen or even added a third hypomethylating agent like decitabine (20 mg/m^2^ intravenously daily for 5 days) or azacytidine (100 mg subcutaneously daily for 7 days) to the former induction regimen. Other partial remission patients converted to granulocyte colony-stimulating factor (G-CSF) priming low dose cytarabine regimen (with G-CSF subcutaneously daily form d1 to d14, cytarabine 10 mg/m^2^ subcutaneously every 12 h from d1 to d14 and aclarubicin 14 mg/m^2^ intravenously daily from d1 to d4) or cephalotoxine ester included regimen (like homoherringtonine 1 mg/m^2^ intravenously daily from d1 to d14 and cytarabine 10 mg/m^2^ subcutaneously every 12 h from d1 to d14) since there are no standard chemotherapy for this situation. No remission patients mostly were given intermediate-dose cytarabine regimens that included purine nucleoside analogues like fludarabine (comprised by fludarabine 30 mg/m^2^ intravenously daily form d2 to d6, cytarabine 2 g/m^2^ intravenously over 4 h starting 4 h after fludarabine and G-CSF subcutaneously daily from d1 to d7) or cladribine (comprising with cladribine 5 mg/m^2^ intravenously daily from d1 to d5, cytarabine 2 g/m^2^ intravenously daily from d1 to d5 and G-CSF subcutaneously daily from d0 to d5), and even one were given HLA fully matched sibling donor stem cell transplantation as rescue. Others no remission patients converted to G-CSF priming low dose cytarabine regimen mentioned above. Few patients were refractory to two consecutive induction treatments and would convert to salvage chemotherapy like the fludarabine or cladribine included regimens mentioned above or allogeneic stem cell transplantation. Most patients would achieve complete remission, and after one or two cycles of consolidation treatment like intermediate-dose cytarabine alone, they would be given stem cell transplantation in case of having a suitable stem cell donor and good remission status, or they would be followed up in out-patient if they are not having a donor or not in good performance status like ECOG score of no more than 2. The conditioning regimen at transplantation were all myeloablative with either total body irradiation of a dose greater than 6 Gray or intravenous busulfan of total dose greater than 6.4 mg/Kg as previous report [32]. So we stratified the conditioning regimen into six categories referring to previous published method rather than basing on intensity [17]. The stem cell transplantation was classified into autosomal stem cell transplantation and allogenetic stem cell transplantation which was further subgrouped into HLA fully matched sibling donor transplantation, HLA haploidentical relative’s donor transplantation and HLA fully matched unrelated donor transplantation. The treatment procedure of enrolled patients were also shown in Fig. 1 of patient flow diagram. The grading of acute graft versus host disease (aGVHD) were according to modified Keystone Criteria [27]. The global severity of chronic GVHD (cGVHD) were classified according to consensus published previously [19].

### Risk classification

Bone marrow hematopoietic cells chromosome examinations were performed in all patients and karyotype results were applied in the risk classification. Some patients underwent new generation sequencing of genes with pivotal prognostic values like NPM1, FLT3, CEBPA, RUNX1, ASXL1 and TP53 [15], and genes with epigenetic modulating function like DMNT3A, TET2, IDH1 or IDH2 [25]. Some patients detected the MLL fusion genes by polymerase chain reaction described before [2]. Patients were classified into favorable, intermediate or adverse risk category according to their cytogenetics and molecular abnormalities as published in 2016 NCCN clinical practice guidelines for AML [26].

### Response criteria

The responses of induction therapy were classified into three status as complete remission (CR), partial remission (PR) and no remission (NR). CR referred to complete relief of all leukemia related symptoms like fever, bleeding, fatigue and so on, as well as parameters of bone marrow blast cells less than 5% level, peripheral neutrophil more than 1000/μl and platelet count more than 100,000/μl. PR referred to complete remission of leukemia related symptoms and substantial decrement of the bone marrow leukemia cells to more than 50% of former level and to less than 25% but above 5% level. NR referred to not fulfil the conditions of CR or PR as those raised above [11]. Those patients couldn’t achieve CR after two induction courses were classified as chemotherapy refractory patients. Patients who died within 7 days of completing each of the two inductive chemotherapies or after 7 days of complications arising from marrow aplasia, usually hemorrhage or sepsis, before any remission status could be ascertained were defined as induction treatment failure.

### Outcomes definition

CR of the primary two inductions were observed at first as a major outcome. With the increment of follow-up duration, the patients’ overall survival time (OS) were calculated from the date of diagnosis of leukemia until death due to any cause and were censored at the last follow up of April 30th, 2023. The progress free survival time (PFS) were calculated in those not refractory patients from the date of achieving CR until loss of CR or death of any cause and were censored at the last follow up. Patents who underwent hematopoietic stem cell transplantation were not censored at the date of transplantation.

### Statistical analysis

The IBM IPSS statistic program V22 were used for analysis. To test factors that predict CR, the χ^2^ test and t test were used for univariate analysis, and the multiple logistic regression model was used for multivariate analysis. The Kaplan-Meier method was used to estimate probabilities of OS and PFS. The log-rank test was applied in univariate analysis and the proportional hazard model of Cox in multi-variate analysis of OS and RFS comparison. All statistical tests were 2 sided and set at 0.05 for significant level.

## Results

### Patient characteristics

**Fig. 2:**
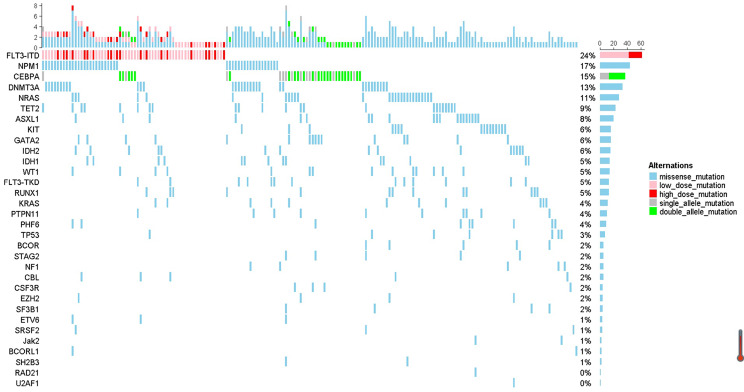
Mutation landscapes of patients with newly diagnosed AML treated with “3+7” induction chemotherapy. (Left) The names of genes which mutation frequency is more than 1. (Right) The frequency of each mutation

There were 219 patients who received idarubicin in their first standard “3+7” induction chemotherapy and 36 patients who received daunorubicin in their first chemotherapy included in this study. The characteristics of patients divided into two groups were listed in Table 1. The median age at onset was 39.5 years old (range, 10–70 years). Fifty percent patients were given the first course of chemotherapy at other blood centers. 236 patients (92.6%) had white blood cell count documented at the time of diagnosis. 235 patients (92.2%) could be assigned to 3 cytogenetic and molecular risk categories. Among them, 82 (34.9%) were classified into favorable group, 102 (43.4%) were classified into intermediate group and 51 (21.7%) in the adverse group. Only 9 patients were secondary AML. All evaluable patients had ECOG score of no more than 3. More percent of patients in the daunorubicin group firstly inducted in other none-tertiary care centers comparing with patients of idarubicin group but without statistical significance (see Table 1). Less proportion of patients with more than 50 × 10^9^/L blood cell count at time of diagnosis placed in the daunorubicin group (see Table 1), since these patients would preferentially select a tertiary care center for first induction and more likely receive idarubicin based regimen. Gene mutation landscape is shown in Fig. 2. FLT3-ITD mutation is most frequently happened in these intensive chemotherapy fit patients.

**Table 1:** The general characteristics of AML patients included in the idarubicin and daunorubicin groups

	IDR group, n	DNR group, n	***P***
Median years of age at onset (range)	40 (10–70)	31 (11–67)	0.382
<20 (%)	17 (7.8)	7 (19.4)	
≥20 and ≤40 (%)	95 (43.4)	15 (41.6)	
>40 and ≤60 (%)	97 (44.3)	11 (30.6)	
≥60 (%)	10 (4.6)	3 (8.3)	0.081
Gender			
Female (%)	96 (43.8)	12 (31.4)	
Male (%)	123 (56.2)	24 (68.6)	0.167
Site of their first chemotherapy course			
Our center (%)	115 (52.5)	13 (36.1)	
Other blood centers (%)	104 (47.5)	23 (63.9)	0.169
ECOG score documented			
0 (%)	26 (12.6)	2 (6.1)	
1 (%)	79 (38.3)	14 (42.4)	
2 (%)	89 (43.2)	14 (42.4)	
3 (%)	12 (5.8)	3 (9.1)	0.617
Secondary AML			
Yes	8 (3.7)	1 (2.8)	
No	209 (96.3)	35 (97.2)	1.000
Median white blood cell count at diagnosis	27.3 × 10^9^/L	17.5 × 10^9^/L	0.428
≤20 × 10^9^/L (%)	94 (41.2)	15 (41.7)	
20–50 × 10^9^/L (%)	37 (18.0)	6 (16.7)	
>50 × 10^9^/L (%)	75 (34.0)	8 (22.2)	
Unknown	13 (6.7)	7 (19.4)	0.035
Cytogenetic risk group			
Favourable (%)	74 (33.8)	8 (22.2)	
Intermediate (%)	90 (41.1)	12 (33.3)	
Adverse (%)	40 (18.3)	11 (30.5)	
Unknown (%)	15 (6.8)	5 (13.9)	0.110

*IDR* idarubucin, *DNR* daunorubicin, *FAB* French-American-British classification

### Response to induction therapy

Of all patients, 158 (61.9%) achieved CR after first course of induction. Idarubicin group had more CR rate than daunorubicin group with marginal significance (*P* = 0.06, see Table 2). 4 NR patients after first course of induction didn’t undergo the second course of chemotherapy and were unevaluable if they are chemotherapy refractory or not. 2 of them died of serious infections post chemotherapy and other 2 of them was bridged to transplantation directly. So, 31 patients eventually turned out to be of chemotherapy refractory. The chemotherapy refractory rates of evaluable idarubicin and daunorubicin group were 10.2% and 25.0% respectively, which were significantly different (*P* = 0.02). The chemotherapy refractory or induction failure numbers of two groups were 24 (11.1%) and 9 (25.7%) respectively with statistically significant difference (*P* = 0.034, see Table 2). The CR rates after first induction and chemotherapy refractory rates in different groups were stratified by ECOG classification, diagnosis of secondary or de novo AML, first treatment site, cytogenetic risk group, age at onset and white blood cell count at diagnosis and listed in Table 3. In de novo AML, favorable risk patients and those of no more than 20 years old, the CR rate of idarubicin based regimen were statistically higher than that of daunorubicin included regimen (*P* = 0.044, 0.043 and 0.023 respectively, see Table 3). In groups of de novo AML, favorable risk patients and patients firstly inducted in our center, the refractory rate of idarubicin included regimen were lower than daunorubicin included regimen significantly (*P* = 0.023, 0.024 and 0.04 respectively).

**Table 2:** Results of induction therapy

	IDR group, n (%)	DNR group, n (%)	OR	***P***
	219	36		
CR by 1 course	142 (65.8)	17 (47.2)	2.061	0.06
CR after 2 courses	193 (88.1)	27 (75.0)	2.474	0.06
Chemotherapy refractory	22 (10.0)	9 (25.0)	2.99	0.02
Chemotherapy Refractory or induction failure by 2 courses of treatment	24 (11.2)	9 (25.0)	2.71	0.03
Converting to transplantation for NR after first induction	2 (0.9)	0 (0)		1.0

*IDR* idarubucin, *DNR* daunorubicin

**Table 3:** CR rates in rows stratified by general characteristics

	CR rate, n (%)			Refractory rate, n (%)		
	IDR group	DNR group	*P*	IDR group	DNR group	*P*
Evaluable patients	219	35		215	35	
**ECOG classification**						
≤1	66 (62.9)	8 (50)	0.326	10 (9.7)	5 (31.2)	0.016
≥2	66 (65.3)	7 (41.2)	0.058	8 (8.1)	4 (23.5)	0.053
**Diagnosis classification**						
Secondary AML	6 (75.0)	1 (100)	1	1 (12.5)	0 (0)	1
De novo AML	139 (64.7)	17 (47.2)	0.044	21 (10.2)	9 (25.7)	0.023
**Cytogenetic risk group**						
Favourable	62 (83.8)	4 (50.0)	0.043	1 (1.4)	2 (25)	0.024
Intermediate	52 (57.8)	5 (41.7)	0.291	12 (13.6)	3 (25)	0.383
Adverse	15 (37.5)	6 (54.5)	0.309	9 (23.1)	3 (27.3)	1.0
Unknown	12 (80.0)	2 (40.0)	0.131	0 (0.0)	1 (20.0)	1.0
**First chemotherapy site**						
Our center	78 (68.4)	5 (38.5)	0.060	10 (8.9)	4 (30.8)	0.04
Other centers	63 (60.0)	12 (52.2)	0.490	12 (11.7)	5 (21.7)	0.196
**Age at onset (years)**						
≤20	12 (70.6)	1 (14.3)	0.023	1 (5.9)	3 (42.9)	0.059
>20 and ≤40	56 (58.3)	11 (73.3)	0.269	12 (12.6)	3 (20.0)	0.428
>40 and ≤60	66 (68.8)	5 (45.5)	0.176	8 (8.6)	3 (27.3)	0.091
>60	7 (70)	0 (0)	0.070	1 (10.0)	0 (0)	1.0
**WBC at diagnosis, ×10** ^**9**^ **/L**						
≤20	53 (56.4)	8 (53.3)	0.825	8 (8.6)	4 (26.7)	0.062
>20 and ≤50	26 (70.3)	4 (66.7)	1.0	4 (11.1)	1 (16.7)	0.557
>50	55 (72.4)	3 (37.5)	0.10	8 (10.8)	3 (37.5)	0.07
Unknown	7 (53.8)	2 (28.5)	0.374	2 (16.6)	1 (14.2)	1.0

*IDR* idarubucin, *DNR* daunorubicin, *FAB* French-American-British classification, *WBC* white blood cell count, *NA* not available for small sample

### Other factors associating with response to induction

The relationships of variables like induction regimen, ECOG classification, AML classification, risk classification, onset age, first induction site, white blood cell count and mutations of DMNT3A, TET2, IDH1, IDH2 with CR after one course of induction or chemotherapy refractory were listed in Table 4. Overall, logistic regression analysis revealed that risk classification was only independent prognostic factor related with achieving CR by one course of induction or chemotherapy refractory. Higher risk group has OR of 0.211 for achieving CR and 5.061 for chemotherapy refractory comparing with lower risk group (*P* = 0.000 and 0.041 respectively).

**Table 4:** Factors that predicted CR after one course of chemotherapy and chemotherapy refractory in all evaluable patients by multi-variate analysis

Variables	CR after first course of chemotherapy		Chemotherapy refractory	
	OR (95% CI)	*P* value	OR (95% CI)	*P* value
Cytogenetic risk group	0.211 (0.094–0.476)	0.000	5.061 (1.071–23.921)	0.041
DNMT3A mutation	0.406 (0.145–1.136)	0.086	1.160 (0.249–5.401)	0.850
WBC count	1.005 (0.999–1.010)	0.112	1.00 (0.993–1.008)	0.972
IDH1 mutation	2.815 (0.618–12.812)	0.181	0.600 (0.063–5.764)	0.658
de novo to secondary AML	0.284 (0.030–2.718)	0.275	0.433 (0.040–4.695)	0.491
TET2 mutation	1.721 (0.546–5.430)	0.354	0.446 (0.049–4.050)	0.473
IDH2 mutation	0.629 (0.184–2.143)	0.458	3.190 (0.776–13.112)	0.108
DNR based induction	1.117 (0.403–3.097)	0.832	2.419 (0.705–8.301)	0.160
Higher ECOG score	1.065 (0.520–2.182)	0.863	0.776 (0.270–2.234)	0.639
Onset age	1.002 (0.976–1.028)	0.890	0.983 (0.944–1.023)	0.395
initially inducted in our center	1.008 (0.496–2.047)	0.983	0.849 (0.297–2.423)	0.759

OR reflected the increased risk of CR for lower cytogenetic risk groups, increasing of one year age or increment of 1 × 10^9^/L WBC count

### Post the initial two induction therapy

Of the unfavorable cytogenetic molecular risk AML patients, 117 (76.4%) underwent stem cell transplantation by the end of follow-up. 97 (82.9%) patients were given transplantation in CR1. 7 patients were given transplantation in CR2. Others undertook the stem cell transplantation as a salvage treatment directly. 36 unfavorable cytogenetic molecular risk patients haven’t been given transplantation by the end of follow-up because of refusing to in 18 patients achieving CR1, relapse within 3 months after achieving CR1 in 6 patients, and chemotherapy refractory in 10 patients, death from consolidation chemotherapy in 2 patients.

### OS and PFS

**Fig. 3:**
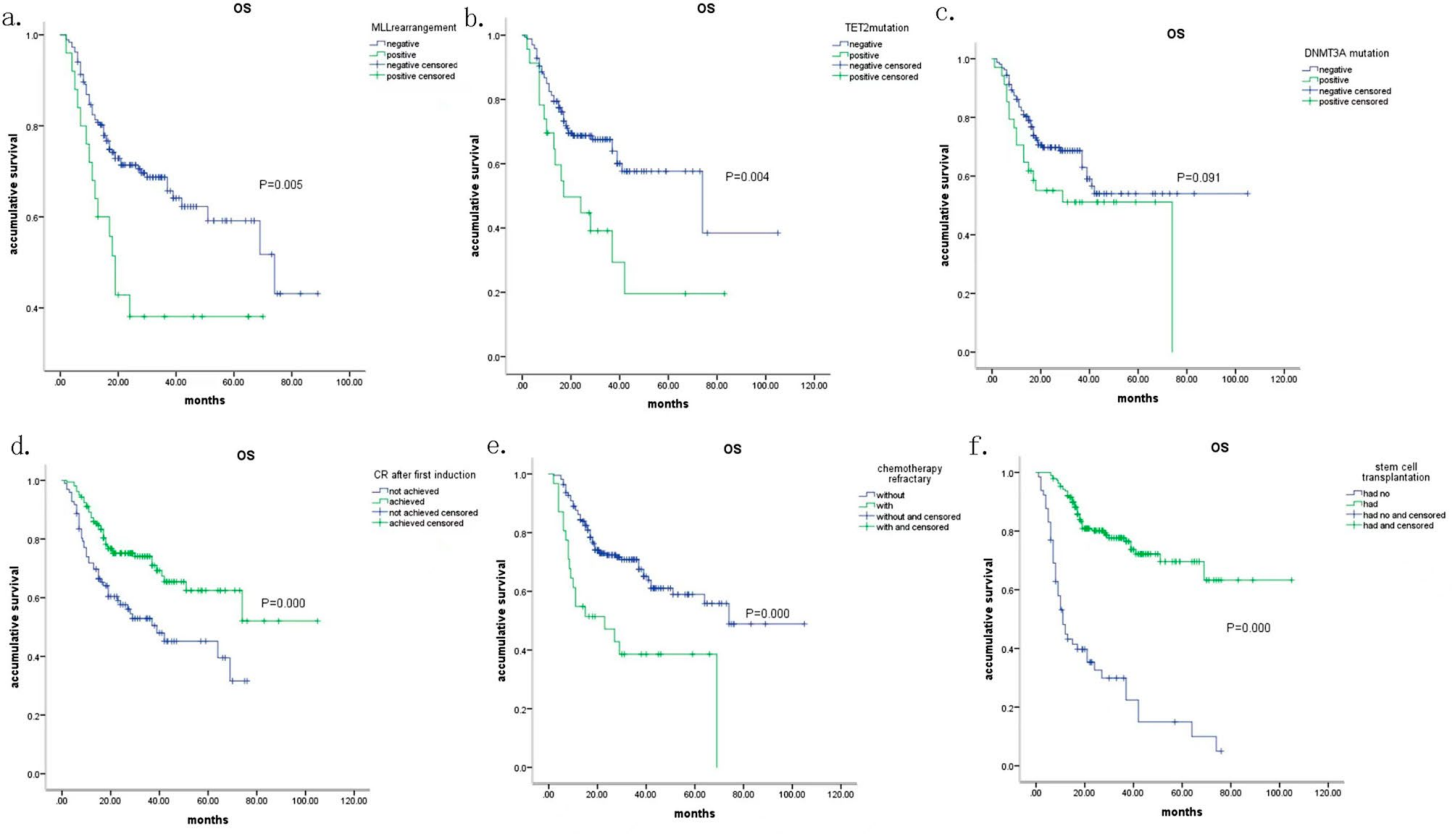
Kaplan Meier curve of OS **a** predicted median survival time for patients without and with MLL rearrangement were 74 and 19 months. **b** predicted median survival time for patients without and with TET2 mutation were 74 and 17 months. **c** predicted median survival time for patients without and with DNMT3A mutation were not reached and 74 months. **d** predicted median survival time for patients not achieving CR and had achieved CR for first induction chemotherapy were 39 months and not reached. **e** predicted median survival time for patients of no chemotherapy refractory and the chemotherapy refractory were 74 and 23 months. **f** predicted median survival time for patients had no stem cell transplantation and had stem cell transplantation 11 months and not reached

With a median follow-up of 34 months, the predicted median OS for all patients observed were 69 months (95% CI 48.4 to 89.5 months). The OS time was significantly corelated with genes for epigenetic modulating function like DMNT3A and TET2, MLL rearrangement, achieving CR after first induction, chemotherapy refractory and having stem cell transplantation (see Fig. 3). By Cox proportional hazard model analysis, not having stem cell transplantation and not achieving CR after first induction were the independent negative prognostic feature for OS (see Table 5), and not chemotherapy refractory, without MLL rearrangement, no TET2 mutation or DMNT3A mutation were the independent favorable prognostic features for OS (see Table 5). For PFS, not having transplantation was independent negative prognostic feature for progress with hazard risk of 4.887 (95% CI 2.763 to 8.664; *P* = 0.000). Not chemotherapy refractory, not having DNMT3A mutation or MLL rearrangement were independent favorable features for PFS with progression risk of 0.286, 0.448 and 0.287 respectively (*P* = 0.012, 0.013 and 0.001). Cytogenetic risk classification, TET2 mutation, onset age, WBC count at diagnosis did not independently relate with either OS or PFS in multivariate analysis.

**Table 5:** Results of the multivariate analysis of independent factors associated with overall survival in our AML patients

	HR	95% CI	***P***
**Stem cell transplantation**			
Had	1		
Haven’t had	7.068	3.907–12.787	0.000
**MLL rearrangement**			
Positive	1		
Negative	0.184	0.088–0.385	0.000
**TET2 mutation**			
Positive	1		
Negative	0.314	0.151–0.655	0.002
**Chemotherapy refractory**			
Yes	1		
No	0.310	0.140–0.686	0.004
**DNMT3A mutation**			
Positive	1		
Negative	0.492	0.255–0.947	0.034
**Initial induction chemotherapy respond**			
CR	1		
No CR	1.883	1.016–3.488	0.044

### Factors associating with survival time post transplantation

Unfavorable cytogenetic molecular risk patients were legible for consolidation of stem cell transplantation and were sorted for further analysis to seek for statistically significant factors associating with long-term outcomes like survive or relapse after transplantation. The median follow-up time post transplantation for these patients was 26 months. CR at transplantation, not MLL rearrangement or TET2 mutation were independently favorable features associating with survive post transplantation (see Table 6). CR at transplantation and not MLL rearrangement were found to be independent favorable factors relating with avoiding relapse post transplantation (see Table 6). In the allogeneic transplanted patients, CR at transplantation, not MLL rearrangement or TET2 mutation and use of ATG were favorable independent features for survival post-transplant (see Table 6). Achieving CR after first induction or CR at time of transplantation, not MLL rearrangement, not DNMT3A mutation or not KRAS mutation were favorable independent factors associating with avoiding relapse after transplantation (see Table 6).

**Table 6:** Results of the multivariate analysis of independent factors associated with survival time and relapse free time post transplantation

	AML patients who received transplantation						AML patients allogenetically tansplantated					
	Death event post transplantation			Relapse post transplantation			Death event post transplantation			Relapse post transplantation		
	HR	95% CI	***P***	HR	95% CI	***P***	HR	95% CI	***P***	HR	95% CI	***P***
**MLL rearrangement**												
Yes	1			1			1			1		
No	0.104	0.037–0.288	0.000	0.120	0.041–0.352	0.000	0.115	0.038–0.348	0.000	0.116	0.020–0.672	0.016
**TET2 mutation**												
Yes	1						1					
No	0.183	0.059–0.566	0.003				0.106	0.031–0.361	0.000			
**Remission at time of transplantation**												
CR	1			1			1			1		
No CR	22.197	6.147–80.16	0.000	15.497	4.212–57.011	0.000	23.719	6.16–91.331	0.000	17.078	1.458–200.1	0.024
**Use of ATG**												
yes							1					
no							3.232	1.232–8.481	0.017			
**Remission to first induction**												
Yes										1		
No										11.233	1.672–75.452	0.013
**DNMT3A mutation**												
Yes										7.648	1.324–44.192	0.023
No										1		
**KRAS mutation**												
Yes										8.965	1.726–46.568	0.009
No										1		

### Adverse events

One hundred and thirty patients underwent initial induction chemotherapy in our blood center and their adverse events were evaluable. The mean duration with a neutrophil count less than 1.0 × 10^9^/L after the initial chemotherapy were 25.3 days for the idarubicin group and 22.2 days for the daunorubicin group (*P* = 0.539). And the mean duration with platelet counts less than 20 × 10^9^/L was 14.4 days for the idarubicin group and 14.7 days for the daunorubicin group (*P* = 0.943). Sepsis occurred in 4 patients in the idarubicin group but none in the daunorubicin group. Early death within 60 days occurred in 1 patient in each chemotherapy group. The most frequently occurred adverse events were pneumonia and febrile neutropenia which accounting for 66.9% cases. Gastrointestinal and upper respiratory infections were other major adverse events. We didn’t find significant difference of adverse events occurrence between two groups (Table 7).

**Table 7:** Adverse events after the start of induction chemotherapy in evaluable patients

	IDA group (n = 116) n (%)	DNR group (n = 14) n (%)	***p***
Sepsis	4 (3.4)	0 (0)	1.0
Early death	1 (0.9)	1 (7.1)	0.205
Febrile neutropenia	35 (30.2)	5 (35.7)	0.703
Pneumonia	43 (37.1)	4 (28.6)	0.571
Gastrointestinal or upper respiratory infection	7 (6.0)	0 (0)	1.0

## Discussion

Our real-world retrospective observation verified again that idarubicin included regimen was superior to daunorubicin included regimen as an initial induction chemotherapy for achieving CR and avoiding chemotherapy resistant. The differences between idarubicin and daunorubicin effects were even statistically obvious when secondary AML was excluded. The results coincided with the former meta-analyses which reported the similar tendency with much larger samples included in the comparison, but fewer stem cell transplantation included in the treatment schedule [1, 20, 35]. Meanwhile, we observed a similar post induction CR rate to that of former clinical trials in idarubicin group [4, 20, 28]. Our CR rate after first induction with daunorubicin was also comparable with that of former reported articles using similar standard “3+7” regimen [6, 36]. Although previous article reported that the 3 days of idarubicin caused more profound myelosuppression than ordinary daunorubincin dosage [22], We didn’t find significant prolongation of neutropenia and thrombocytopenia after induction treatment of idarubicin comparing with daunorubicin. The result suggested both regimens are biological dose equivalent. As to adverse events, Pneumonia and febrile neutropenia almost equally happened in our two groups. But we found more cases of sepsis and gastrointestinal or upper respiratory infections in idarubicin group without statistical significance. This is also coinside with previous reports.

Although idarubicin showed its advantage in induction of CR and avoiding chemotherapy resistance, it was not verified to be statistically significant in multivariate analysis. Some factors were considerablely different between idarubicin and daunorubicin based regimen in our collection of retrospective data. More percent of favourable and less percent of adverse cytogenetic risk classified patients were found in idarubicin group (see Table 1). To our interest, lower cytogenetic molecular risk grade was found to be the only statistically independent favorable factor to predict initial induction CR and avoiding chemotherapy refractory. Such relationship between risk classification and CR induction was also implied in the past reports which concluded cytogenetics were key factor associated with more probability of early and deep responses whatever in adults with AML or the elder with AML [10, 29]. The relationship looks reasonable since some critical mutations which deciding AML risk classification closely related with the neoplasm clone evolution (such as BCR-ABL1 fusion, complex karyotye) and cell apoptosis abnormality (such as TP53 mutaiton). Such mutations may influenced chemotherapy sensitivity and their inbalance of distribution in treatment groups influenced CR rates profoundly.

As to OS and PFS, our results verified that having stem cell transplantation or not chemotherapy refractory not only independently favored OS, but also independently favored PFS. Other previous studies on AML patients either of all cytogenetic risks or of the senior aged also found having transplantation an independent favorable factor relating long-term survival [14, 33]. On one hand, transplantation procedure not only more deeply reduced the burden of leukemia cells, but also may deliver a long lasting allo-genetic anti-tumor effect for majority of AML patients. On the other hand, chemotherapy refractory reflects the virtue of leukemia cells to resist chemotherapy drugs and the probability of leukemia residue lasting. So, it’s not difficult to understand their relationships with OS and PFS.

Our multivariate analysis further showed MLL rearrangement and DNA methylating-related genes’ mutations to be the negative features associating with OS and PFS. MLL rearrangement leads to the fusion of MLL with transcriptional activators like AF4, AF9, AF10 and ENL [13]. These fusion proteins overexpresses MLL target genes, including HOXA9 and MEISI, which are required for hemopoietic stem cell self-renewal [24]. So MLL rearrangement may support leukemia transformation and negatively relate with our patient’s survival and disease relapse. DNMT3A mutation was also the independent negative factor relating with OS and PFS in our patients. DNMT3A is responsible for the de novo methylation of DNA. DNMT3A mutation was thought to be a loss of function mutation [18]. DNMT3A mutation was found to be present in highly purified hematopoietic stem cells in AML patients, and stem cells bearing DNMT3A mutation showed a multilineage repopulation advantage over stem cells without DNMT3A mutation [34]. So DNMT3A mutation appears to be the representatives of preleukemic cells that eventually evolves in AML. This feature of DNMT3A mutation may explain the relationship of this mutation with inferior OS and PFS.

MLL rearrangement was again verified to be the independent relative factors with overall survival post transplantation and relapse free survival post transplantation. The result suggested the MLL rearrangement bear a rather closely relationship with disease development. To achieve CR at time of transplantation, rather not chemotherapy refractory, is another independent factor correlated with both good survival and less relapse post transplantation. This result suggested comparing with having a chemotherapy resistant feature, to achieve a temporarily leukemia free status before transplantation is more important to survive for patients with desired transplantation.

In this observation, we verified some obvious relative factors with CR induction and long-term survival of AML patients. But the sample was imbalanced between the groups of idarubicin and daunorubicin. In fact, in the real-world practices more and more AML patients tend to be given idarubicin as an initial induction treatment in a tertiary center. It may cause the sample variation larger in our daunorubicin group and limit the sample presentational power. Additionally, the median follow-up time of our patients are still limited which cause the longer outcome results are not available in most patients, and we cannot compare the 5-year OS and PFS between subgroups. Despite the limitations above, we still could conclude that beside clinical factors, like stem cell transplantation, CR at transplantation and chemotherapy refractory, MLL rearrangement and DNA methylating related gene’s mutations, like DNMT3A mutation and TET2 mutation, were the equally critical features to predict long-term survival for AML.

## Conclusion

Our clinical observation supported that traditional “3+7” chemotherapy regimen with idarubicin plays better in CR induction than that with daunorubicin. But the patients long-term survival related with clinical aspects, like having stem cell transplantation and achieving CR at transplantation, as well as genetic alterations equally, like MLL rearrangement and DNA methylating related genes’ mutations.

## Data Availability

All data generated or used during the study appeared in the submitted article.

## References

[1] AML Collaborative Group. A systematic collaborative overview of randomized trials comparing idarubicin with daunorubicin (or other anthracyclines) as induction therapy for acute myeloid leukaemia. Br J Haematol. 1998;103(1):100–09. (A collaborative overview, using individual patient data, has been performed to compare idarubicin versus daunorubicin or other anthracyclines, when used with cytosine arabinoside as induction chemotherapy for newly diagnosed acute myeloid leukaemia. There were 1052 patients in five trials versus daunorubicin, 100 in one trial versus doxorubicin, and 745 in one trial versus zorubicin. In the trials of idarubicin versus daunorubicin, early induction failures were similar with the two treatments (20% idarubicin v 18% daunorubicin: P = 0.4), but after day 40 the later induction failures were fewer with idarubicin (17% v 29%: P < 0.0001). Therefore complete remission rates were higher with idarubicin (62% v 53%; P = 0.002). Among remitters, fewer of the patients allocated to idarubicin relapsed (P = 0.008) but slightly more died in remission, leading to a non-significant benefit (P = 0.07) in disease-free survival. Overall survival in these five trials was significantly better with idarubicin than with daunorubicin (13% v 9% alive at 5 years; P = 0.03). There was a trend (P = 0.006 for remission rate) for the benefit of idarubicin over daunorubicin to decrease with increasing age. There were no significant differences in outcome in the small trial comparing idarubicin versus doxorubicin, or in the large trial comparing idarubicin versus zorubicin. The induction regimens based on idarubicin achieved, in the particular circumstances of the trials reviewed here, better remission rates and better overall survival than those based on daunorubicin.) 9792296

[2] Andersson A, et al. Paired multiplex reverse-transcriptase polymerase chain reaction (PMRT-PCR) analysis as a rapid and accurate diagnostic tool for the detection of MLL fusion genes in hematologic malignancies. Leukemia. 2001;15(8):1293–300. (The MLL gene in chromosome band 11q23 is frequently rearranged in acute lymphoblastic and acute myeloid leukemias. To date, more than 50 different chromosomal regions are known to participate in translocations involving 11q23, many of which affect MLL. The pathogenetically important outcome of these rearrangements is most likely the creation of a fusion gene consisting of the 5’ part of the MLL gene and the 3’ end of the partner gene. Although abnormalities of the MLL gene as such are generally associated with poor survival, recent data suggest that the prognostic impact varies among the different fusion genes generated. Hence, detection of the specific chimeric gene produced is important for proper prognostication and clinical decision making. We have developed a paired multiplex reverse-transcriptase polymerase chain reaction analysis to facilitate a rapid and accurate detection of the most frequent MLL fusion genes in adult and childhood acute leukemias. To increase the specificity, two sets of primers were designed for each fusion gene, and these paired primer sets were run in parallel in two separate multiplex one-step PCR reactions. Using the described protocol, we were able to amplify successfully, in one single assay, the six clinically relevant fusion genes generated by the t(4;11)(q21;q23) [MLL/AF4], t(6;11)(q27;q23) [MLL/AF6], t(9;11)(p21-22;q23) [MLL/AF9], t(10;11)(p11-13;q23) [MLL/AF10], t(11;19)(q23;p13.1) [MLL/ELL], and t(11;19)(q23; p13.3) [MLL/ENL] in cell lines, as well as in patient material.) 11480574 10.1038/sj.leu.2402189

[3] Begna KH, et al. European LeukemiaNet-defined primary refractory acute myeloid leukemia: the value of allogeneic hematopoietic stem cell transplant and overall response. Blood Cancer J. 2022;12(1):7. (We sought to appraise the value of overall response and salvage chemotherapy, inclusive of allogeneic hematopoietic stem cell transplant (AHSCT), in primary refractory acute myeloid leukemia (prAML). For establishing consistency in clinical practice, the 2017 European LeukemiaNet (ELN) defines prAML as failure to attain CR after at least 2 courses of intensive induction chemotherapy. Among 60 consecutive patients (median age 63 years) correspondent with ELN-criteria for prAML, salvage was documented in 48 cases, 30/48 (63%) being administered intensive chemotherapy regimens and 2/48 consolidated with AHSCT as first line salvage. 13/48 (27%) attained response: CR, 7/13 (54%), CRi, 2/13 (15%), MLFS, 4/13 (31%). The CR/CRi rate was 9/48 (19%), with CR rate of 7/48 (15%). On univariate analysis, intermediate-risk karyotype was the only predictor of response (44% vs 17% in unfavorable karyotype; P = 0.04). Administration of any higher-dose (>1 g/m(2)) cytarabine intensive induction (P = 0.50), intensive salvage chemotherapy (P = 0.72), targeted salvage (FLT3 or IDH inhibitors) (P = 0.42), greater than 1 salvage regimen (P = 0.89), age < 60 years (P = 0.30), and de novo AML (P = 0.10) did not enhance response achievement, nor a survival advantage. AHSCT was performed in 12 patients with (n = 8) or without (n = 4) CR/CRi/MLFS. 1/2/5-year overall survival (OS) rates were 63%/38%/33% in patients who received AHSCT (n = 12) vs 27%/0%/0% in those who achieved CR/CRi/MLFS but were not transplanted (n = 5), vs 14%/0%/0% who were neither transplanted nor achieved CR/CRi/MLFS (n = 43; P < 0.001); the median OS was 18.6, 12.6 and 5.6 months, respectively. Although CR/CRi/MLFS bridged to AHSCT (n = 8), appeared to manifest a longer median OS (20 months), vs (13.4 months) for those with no response consolidated with AHSCT (n = 4), the difference was not significant P = 0.47. We conclude AHSCT as indispensable for securing long-term survival in prAML (p = 0.03 on multivariate analysis), irrespective of response achievement.) 35039473 10.1038/s41408-022-00606-8 PMC8764050

[4] Beksac M, et al. Randomised unicenter trial for comparison of three regimens in de novo adult acute nonlymphoblastic leukaemia. Med Oncol. 1998;15(3):183–90. (Various regimens have been explored in the treatment of acute nonlymphoblastic leukaemia (AML), but so far none has been shown to be superior. Here we report on a comparison of three widely used protocols defined by Berman (Group 1), MRC AML 10 (Group 2), and Arlin (Group 3). Group 1 includes cytosine arabinoside (Ara-C) (100 mg/m2/d, days 1-7) and idarubicin (Ida) (12 mg/m2/d, days 1-3) for induction, and Ara-C (200 mg/m2/d, days 1-6) and Ida (15 mg/m2/d, day 1) twice for consolidation. Group 2 includes Ara-C (200 mg/m2/d, days 1-10), daunorubicin (Dnc) (50 mg/m2/d, days 1, 3, 5) and etoposide (VP16) (100 mg/m2/d, days 1-5) for induction. The first consolidation therapy consisted of the same schedule except for Ara-C given on days 1-8. The second consolidation regimen consisted of Ara-C (200 mg/m2/d, days 1-8), VP16 (100 mg/m2/d, days 1-5) and amsacrine (100 mg/m2/d, days 1-5). Mitoxantrone (Mitox) (10 mg/m2/d, days 1-5) and Ara-C (200 mg/m2/d, days 1-3) were given as the third consolidation therapy. Group 3 was identical to Group 1 except for Ida being replaced with Mitox. During the study period 99 patients were enrolled and 34 were allocated randomly to Group 1, 36 to Group 2, and 29 to Group 3. Except for age distribution all patients’ characteristics were similar between the groups. As there were more elderly patients in Group 1, time to complete remission (CR) was longer in this group as they needed more second induction. Induction deaths were 9.7%, 12.9% and 14.8% in Groups 1, 2 and 3, respectively. Patients in Group 2 received a higher amount of Ara-C compared with the other groups (P < 0.001). After a median follow-up period of 45 months (1-67 for survivors) an advantage in Group 1 was observed. Relapse-free survival (RFS) was better in Group 1 (P = 0.014) at 3 years. Fourteen of the patients were transplanted (11 allografts, 3 autografts). When patients with transplants were excluded, overall survival was longer in Group 1 both at 3 years and 5 years (P = 0.05). In conclusion, despite patient advanced age and lower dose of Ara-C, the idarubicin-containing treatment was superior to the other regimens.) 9819795 10.1007/BF02821937

[5] Berkman AM, et al. Long-term outcomes among adolescent and young adult survivors of acute leukemia: a surveillance, epidemiology, and end results analysis. Cancer Epidemiol Biomarkers Prev. 2022;31(6):1176–84. (BACKGROUND: There is a growing population of adolescent and young adult (AYA, age 15–39 years) acute leukemia survivors in whom long-term mortality outcomes are largely unknown. METHODS: The current study utilized the Surveillance, Epidemiology, and End Results (SEER) registry to assess long-term outcomes of AYA acute leukemia 5-year survivors. The impact of diagnosis age, sex, race/ethnicity, socioeconomic status, and decade of diagnosis on long-term survival were assessed utilizing an accelerated failure time model. RESULTS: A total of 1,938 AYA acute lymphoblastic leukemia (ALL) and 2,350 AYA acute myeloid leukemia (AML) survivors diagnosed between 1980 and 2009 were included with a median follow-up of 12.3 and 12.7 years, respectively. Ten-year survival for ALL and AML survivors was 87% and 89%, respectively, and 99% for the general population. Survival for AYA leukemia survivors remained below that of the age-adjusted general population at up to 30 years of follow-up. Primary cancer mortality was the most common cause of death in early survivorship with noncancer causes of death becoming more prevalent in later decades of follow-up. Male AML survivors had significantly worse survival than females (survival time ratio: 0.61, 95% confidence interval: 0.45-0.82). CONCLUSIONS: AYA leukemia survivors have higher mortality rates than the general population that persist for decades after diagnosis. IMPACT: While there have been improvements in late mortality, long-term survival for AYA leukemia survivors remains below that of the general population. Studies investigating risk factors for mortality and disparities in late effects among long-term AYA leukemia survivors are needed.) 35553621 10.1158/1055-9965.EPI-21-1388 PMC9179079

[6] Berman E, et al. Results of a randomized trial comparing idarubicin and cytosine arabinoside with daunorubicin and cytosine arabinoside in adult patients with newly diagnosed acute myelogenous leukemia. Blood. 1991;77(8):1666–74. (4’-Demethoxydaunorubicin (idarubicin [IDR]) is a new anthracycline that differs from its parent compound by the deletion of a methoxy group at position 4 of the chromophore ring. This minor structural modification results in a more lipophilic compound with a unique metabolite that has a prolonged plasma half-life as well as in vitro and in vivo antileukemia activity. To determine its activity in acute myelogenous leukemia (AML), 130 consecutive adult patients between the ages of 16 and 60 with newly diagnosed disease were randomized in a single institution study to receive either IDR in combination with cytosine arabinoside (Ara-C) or standard therapy with daunorubicin (DNR) and Ara-C. The trial was analyzed using the O’Brien-Fleming multiple testing design that allowed for periodic inspection of the data at specific patient accession points. After accrual of 60 patients per arm, analysis showed that patients who received IDR/Ara-C had a superior response compared with those who received standard therapy: 48 of 60 patients (80%) achieved complete remission on the former arm compared with 35 of 60 patients on the latter (58%, P =.005). Logistic regression analysis of factors associated with complete response indicated that treatment with IDR/Ara-C offered a significant advantage to patients who presented with a high initial white blood cell count compared with treatment with DNR/Ara-C. The degree of marrow aplasia was approximately the same on each arm as was nonhematologic toxicity. Overall survival for patients on the IDR/Ara-C arm was 19.5 months compared with 13.5 months on the DNR/Ara-C arm (P =.025) at a median follow-up of 2.5 years. We conclude that IDR/Ara-C can effectively replace standard therapy with DNR/Ara-C in adult patients less than age 60 with newly diagnosed AML.) 2015395

[7] Berman E, McBride M. Comparative cellular pharmacology of daunorubicin and idarubicin in human multidrug-resistant leukemia cells. Blood. (1992). 79 (12):3267–73. (We examined the effect of daunorubicin (DNR), the new anthracycline derivative idarubicin (IDR), and verapamil on two leukemia cell lines that displayed the multidrug resistant (MDR) phenotype and used laser flow cytometry to quantitate intracellular anthracycline content. The vinblastine-resistant human lymphoblastic leukemia cell line CEM-VBL demonstrated minimal DNR uptake; simultaneous incubation with verapamil and DNR increased intracellular DNR uptake fourfold. IDR uptake was 10 times more rapid in these cells and simultaneous incubation with IDR and verapamil resulted in only a 1.2-fold increase of intracellular IDR. Similar results were observed in the vincristine-resistant human myeloid leukemia cell line HL-60/RV+. Intracellular retention of DNR and IDR was also measured in each cell line. In CEM-BVL cells, 38% of the original DNR concentration remained after a 2-hour resuspension in fresh medium compared with 71% of the original IDR concentration. In HL-60/RV+ cells, 36% of the DNR concentration remained compared with 51% of the IDR concentration. After incubation of CEM-VBL and HL-60/RV+ cells with DNR for 1 hour followed by resuspension in fresh medium plus verapamil, intracellular DNA retention increased 5- and 5.2-fold, respectively. However, incubation of these cells for 1 hour with IDR followed by resuspension in fresh medium plus verapamil resulted in only a 1.6- and 2.4-fold increase in intracellular IDR retention. Lastly, clonogenic experiments were performed to correlate intracellular anthracycline content with cytotoxicity. DNR alone had a minimal effect on the clonogenic growth of CEM-VBL cells, whereas the combination of DNR plus verapamil resulted in approximately 80% growth inhibition. However, incubation of these cells with IDR alone resulted in greater than 95% growth inhibition. These results suggest that IDR may be more effective than DNR in leukemia cells that display the MDR phenotype.) 1596567

[8] Bloomfield CD, et al. Time to repeal and replace response criteria for acute myeloid leukemia? Blood Rev. 2018;32(5):416–25. (The International Working Group (IWG) response criteria for acute myeloid leukemia, published in 2003, have remained the standard by which the efficacy of new drugs is measured in clinical trials. Over the last decade, concepts related to treatment response have been challenged by several factors; for example, the dissociation between early clinical response and survival outcome in older patients, the recognition that epigenetic and newer differentiating-agent therapies may produce delayed responses and also hematologic improvement/transfusion independence without a morphologic response, and evidence that remissions without minimal (or measurable) residual disease (MRD) may result in outcomes superior to those of morphologic remissions with persistent MRD. The evolving role of MRD status as a potential surrogate for predicting long-term survival has enhanced the clinical need to standardize and incorporate emerging technologies that enable deeper responses beyond those recognized by the IWG, and to pre-emptively identify patients at risk of early relapse. The potential for therapeutic interventions to erase MRD and alter the natural history represents an important and open research question. Reviewed here are some of the implications and challenges associated with establishing and incorporating new treatment response criteria, initially into clinical research, and eventually into real-world practice.) 29706486 10.1016/j.blre.2018.03.006

[9] Bonnet D, Dick JE. Human acute myeloid leukemia is organized as a hierarchy that originates from a primitive hematopoietic cell. Nat Med. (1997). 3 (7):730–37. (On the subject of acute myeloid leukemia (AML), there is little consensus about the target cell within the hematopoietic stem cell hierarchy that is susceptible to leukemic transformation, or about the mechanism that underlies the phenotypic, genotypic and clinical heterogeneity. Here we demonstrate that the cell capable of initiating human AML in non-obese diabetic mice with severe combined immunodeficiency disease (NOD/SCID mice) - termed the SCID leukemia-initiating cell, or SL-IC - possesses the differentiative and proliferative capacities and the potential for self-renewal expected of a leukemic stem cell. The SL-ICs from all subtypes of AML analyzed, regardless of the heterogeneity in maturation characteristics of the leukemic blasts, were exclusively CD34++ CD38-, similar to the cell-surface phenotype of normal SCID-repopulating cells, suggesting that normal primitive cells, rather than committed progenitor cells, are the target for leukemic transformation. The SL-ICs were able to differentiate in vivo into leukemic blasts, indicating that the leukemic clone is organized as a hierarchy.) 9212098 10.1038/nm0797-730

[10] Cancer and Leukemia Group B 8461, et al. Pretreatment cytogenetics add to other prognostic factors predicting complete remission and long-term outcome in patients 60 years of age or older with acute myeloid leukemia: results from Cancer and Leukemia Group B 8461. Blood. 2006;108(1):63–73. (We investigated the relative prognostic significance of cytogenetics in 635 adult acute myeloid leukemia (AML) patients 60 years of age or older treated on front-line protocols. Classification trees and tree-structured survival analysis (TSSA) were used to identify important cytogenetic groups, and their prognostic significance was then assessed in multivariable analysis (MVA). Overall, 48.5% achieved complete remission (CR); 6.6% survived at 5 years. Complex karyotypes with at least 3 abnormalities (complex > or = 3) and a group including “rare aberrations” predicted lower CR rates (25% and 30%) versus other patients (56%). Compared with complex > or = 3, the odds of CR were significantly higher for noncomplex karyotypes without rare aberrations on MVA. Cytogenetically, complex > or = 5 predicted inferior disease-free survival on TSSA, remaining significant on MVA together with white blood cell count (WBC), sex, and age. For survival, complex > or = 5, rare aberrations, and core-binding factor (CBF) abnormalities were prognostic (P <.001), with 5-year survivals of 0%, 0%, and 19.4%, respectively, and 7.5% for remaining patients. Together with WBC, marrow blasts, sex, and age, the cytogenetic groups remained significant on MVA. In conclusion, pretreatment cytogenetics adds to other prognostic factors in older AML patients. Patients with complex > or = 5 appear to benefit minimally from current treatment and are better suited for investigational therapy or supportive care.) 10.1182/blood-2005-11-4354 PMC1895823 16522815

[11] Cheson BD, et al. Revised recommendations of the International Working Group for Diagnosis, standardization of response criteria, treatment outcomes, and reporting standards for therapeutic trials in acute myeloid leukemia. J Clin Oncol. 2003;21(24):4642–49. (An International Working Group met to revise the diagnostic and response criteria for acute myelogenous leukemia originally published in 1990, as well as to provide definitions of outcomes and reporting standards to improve interpretability of data and comparisons among trials. Since the original publication, there have been major advances in our understanding of the biology and molecular genetics of acute leukemia that are clinically relevant and warrant incorporation into response definitions. Differences from the 1990 recommendations included a category of leukemia-free state, new criteria for complete remission, including cytogenetic and molecular remissions and remission duration. Storage of viable blasts for correlative studies is important for future progress in the therapy of these disorders.) 14673054 10.1200/JCO.2003.04.036

[12] Chou SC, et al. Prognostic implication of gene mutations on overall survival in the adult acute myeloid leukemia patients receiving or not receiving allogeneic hematopoietic stem cell transplantations. Leuk Res. 2014;38(11):1278–84. (Several gene mutations have been shown to provide clinical implications in patients with acute myeloid leukemia (AML). However, the prognostic impact of gene mutations in the context of allogeneic hematopoietic stem cell transplantation (allo-HSCT) remains unclear. We retrospectively evaluated the clinical implications of 8 gene mutations in 325 adult AML patients; 100 of them received allo-HSCT and 225 did not. The genetic alterations analyzed included NPM1, FLT3-ITD, FLT3-TKD, CEBPA, RUNX1, RAS, MLL-PTD, and WT1. In patients who did not receive allo-HSCT, older age, higher WBC count, higher lactate dehydrogenase level, unfavorable karyotype, and RUNX1 mutation were significantly associated with poor overall survival (OS), while CEBPA double mutation (CEBPA(double-mut)) and NPM1(mut)/FLT3-ITD(neg) were associated with good outcome. However, in patients who received allo-HSCT, only refractory disease status at the time of HSCT and unfavorable karyotype were independent poor prognostic factors. Surprisingly, RUNX1 mutation was an independent good prognostic factor for OS in multivariate analysis. The prognostic impact of FLT3-ITD or NPM1(mut)/FLT3-ITD(neg) was lost in this group of patients receiving allo-HSCT, while CEBPA(double-mut) showed a trend to be a good prognostic factor. In conclusion, allo-HSCT can ameliorate the unfavorable influence of some poor-risk gene mutations in AML patients. Unexpectedly, the RUNX1 mutation showed a favorable prognostic impact in the context of allo-HSCT. These results need to be confirmed by further studies with more AML patients.) 25260824 10.1016/j.leukres.2014.08.012

[13] Daser A, Rabbitts TH. Extending the repertoire of the mixed-lineage leukemia gene MLL in leukemogenesis. Genes Dev. 2004;18(9):965–74. 15132992 10.1101/gad.1195504

[14] Devillier R, et al. In-depth time-dependent analysis of the benefit of allo-HSCT for elderly patients with CR1 AML: a FILO study. Blood Adv. 2022;6(6):1804–12. (The benefit of allogeneic hematopoietic stem cell transplantation (allo-HSCT) for patients with acute myeloid leukemia (AML) aged >60 years remains a matter of debate, notably when performed in first complete remission (CR1). To clarify this issue, the French Innovative Leukemia Organization (FILO) performed a 10-year real-world time-dependent analysis. The study enrolled patients between 60 and 70 years of age with AML in CR1 after intensive chemotherapy with intermediate (IR) or unfavorable (UR) risk according to the European LeukemiaNet (ELN) 2010 classification. The impact of allo-HSCT was analyzed through three models: (1) time-dependent Cox; (2) multistate for dynamic prediction; and (3) super landmark. The study enrolled 369 (73%) IR and 138 (27%) UR patients with AML, 203 of whom received an allo-HSCT. Classical multivariate analysis showed that allo-HSCT significantly improved relapse-free survival (RFS; hazard ratio [HR] [95% confidence interval (CI)], 0.47 [0.35-0.62]; P <.001) and overall survival (OS; HR [95% CI], 0.56 [0.42-0.76]; P <.001), independently of the ELN risk group. With the multistate model, the predicted 5-year probability for IR and UR patients to remain in CR1 without allo-HSCT was 8% and 1%, respectively. Dynamic predictions confirmed that patients without allo-HSCT continue to relapse over time. Finally, the super landmark model showed that allo-HSCT significantly improved RFS (HR [95% CI], 0.47 [0.36-0.62]; P <.001) and OS (HR [95% CI], 0.54 [0.40-0.72]; P <.001). allo-HSCT in CR1 is reported here as significantly improving the outcome of fit older patients with AML. Long-term RFS without allo-HSCT is very low (<10%), supporting allo-HSCT as being the best curative option for these patients.) 34525180 10.1182/bloodadvances.2021004435 PMC8941467

[15] Dohner H, et al. Acute myeloid leukemia. N Engl J Med 2015;373:1136–52. 26376137 10.1056/NEJMra1406184

[16] Heinicke T, et al. Allogeneic hematopoietic stem cell transplantation improves long-term outcome for relapsed AML patients across all ages: results from two East German Study Group Hematology and Oncology (OSHO) trials. Ann Hematol. 2021;100(9):2387–98. (Relapse of acute leukemia is a frequent complication with uncertain outcome and poorly defined risk factors. From 1621 patients entered into two prospective clinical trials (AML02; n = 740 and AML04; n = 881), 74.2% reached complete remission (CR) 1 after induction(s) and 59 patients after additional induction +/- hematopoietic cell transplantation (HCT). Of the non-refractory patients, 48.4% with a median age of 63 (range 17-85) years relapsed. Relapses occurred within 6 months after CR in 46.5%, between 7 and 18 months in 38.7%, and after 18 months in 14.8% of patients. Relapse treatment resulted in CR2 in 39% of patients depending upon age (54.5% of </= 60 and 28.6% of > 60 years), duration of CR1, and treatment of relapse. Overall survival (OS) was 10.9 (7.4-16.2) %, but OS after HCT +/- intensive chemotherapy (ICT) was 39.3% (31.8-48.6) at 5 years and not different in younger and older patients. Donor lymphocyte infusion +/- chemotherapy and ICT alone resulted only in OS of 15.4% and of 5%, respectively. Independent favorable factors for OS were long CR1 duration, and HCT, while non-monosomal disease was beneficial for OS in elderly patients. Leukemia-free survival [LFS; 24.9 (19.5-31.7) % at 10 years] was affected by similar risk factors. In a competing risk model, the relapse incidence at 5 years was 53.5 +/- 3.5% and the non-relapse mortality rate 21.7 +/- 2.9%. Lower relapse incidence was observed in patents with HCT, long CR1 duration, and female gender. Risk factors for non-relapse mortality were HCT in younger and type of AML in elderly patients. In conclusion, allogeneic HCT +/- IC improved the results in relapsed AML in younger and elderly patients. Increasing CR2 rates and HCT frequency will be the challenge for the next years. Relapse of the disease remains the major problem.) 34232360 10.1007/s00277-021-04565-1 PMC8357692

[17] Hirabayashi S, et al. Personalized prediction of overall survival in patients with AML in non-complete remission undergoing allo-HCT. Cancer Med. 2021;10(13):4250–68. (Allogenic hematopoietic stem cell transplantation (allo-HCT) is the standard treatment for acute myeloid leukemia (AML) in non-complete remission (non-CR); however, the prognosis is inconsistent. This study aimed to develop and validate nomograms and a web application to predict the overall survival (OS) of patients with non-CR AML undergoing allo-HCT (cord blood transplantation [CBT], bone marrow transplantation [BMT], and peripheral blood stem cell transplantation [PBSCT]). Data from 3052 patients were analyzed to construct and validate the prognostic models. The common significant prognostic factors among patients undergoing allo-HCT were age, performance status, percentage of peripheral blasts, cytogenetic risk, chemotherapy response, and number of transplantations. The conditioning regimen was a significant prognostic factor only in patients undergoing CBT. Compared with cyclophosphamide/total body irradiation, a conditioning regimen of >/=3 drugs, including fludarabine, with CBT exhibited the lowest hazard ratio for mortality (0.384; 95% CI, 0.266-0.554; p < 0.0001). A conditioning regimen of >/=3 drugs with CBT also showed the best leukemia-free survival among all conditioning regimens. Based on the results of the multivariable analysis, we developed prognostic models showing adequate calibration and discrimination (the c-indices for CBT, BMT, and PBSCT were 0.648, 0.600, and 0.658, respectively). Our prognostic models can help in assessing individual risks and designing future clinical studies. Furthermore, our study indicates the effectiveness of multi-drug conditioning regimens in patients undergoing CBT.) 34132501 10.1002/cam4.3920 PMC8267144

[18] Holz-Schietinger C, et al. Mutations in DNA methyltransferase (DNMT3A) observed in acute myeloid leukemia patients disrupt processive methylation. J Biol Chem. 2012;287(37):30941–51. (DNA methylation is a key regulator of gene expression and changes in DNA methylation occur early in tumorigenesis. Mutations in the de novo DNA methyltransferase gene, DNMT3A, frequently occur in adult acute myeloid leukemia patients with poor prognoses. Most of the mutations occur within the dimer or tetramer interface, including Arg-882. We have identified that the most prevalent mutation, R882H, and three additional mutants along the tetramer interface disrupt tetramerization. The processive methylation of multiple CpG sites is disrupted when tetramerization is eliminated. Our results provide a possible mechanism that accounts for how DNMT3A mutations may contribute to oncogenesis and its progression.) 22722925 10.1074/jbc.M112.366625 PMC3438927

[19] Jagasia MH, et al. National Institutes of Health Consensus Development Project on criteria for clinical trials in Chronic Graft-versus-Host Disease: I. The 2014 Diagnosis and Staging Working Group report. Biol Blood Marrow Transplant. 2015;21(3):389–401e381. (The 2005 National Institutes of Health (NIH) Consensus Conference proposed new criteria for diagnosing and scoring the severity of chronic graft-versus-host disease (GVHD). The 2014 NIH consensus maintains the framework of the prior consensus with further refinement based on new evidence. Revisions have been made to address areas of controversy or confusion, such as the overlap chronic GVHD subcategory and the distinction between active disease and past tissue damage. Diagnostic criteria for involvement of mouth, eyes, genitalia, and lungs have been revised. Categories of chronic GVHD should be defined in ways that indicate prognosis, guide treatment, and define eligibility for clinical trials. Revisions have been made to focus attention on the causes of organ-specific abnormalities. Attribution of organ-specific abnormalities to chronic GVHD has been addressed. This paradigm shift provides greater specificity and more accurately measures the global burden of disease attributed to GVHD, and it will facilitate biomarker association studies.) 25529383 10.1016/j.bbmt.2014.12.001 PMC4329079

[20] Li X, Xu S, Tan Y, Chen J. The effects of idarubicin versus other anthracyclines for induction therapy of patients with newly diagnosed leukaemia. Cochrane Database Syst Rev 2015, Issue 6. Art. No.: CD010432. (BACKGROUND: Anthracycline combined with cytarabine has been the standard for of evidence), CR rate (eight studies, 2411 patients; RR 0.97, 95% CI 0.92 to 1.03, P = 0.32;moderate quality of evidence), the risks of death on induction therapy (five studies, 2055 patients; RR 1.10, 95% CI 0.88 to 1.38, P = 0.39; moderate quality of evidence) and relapse (three studies, 328 patients; RR 0.99, 95% CI 0.80 to 1.22, P = 0.89; moderate quality of evidence). There was no evidence for difference in the risks of grade 3/4 cardiac toxicity (one study, 160 patients; RR 0.67, 95% CI 0.11 to 3.88, P = 0.65; low quality of evidence) and other grade 3/4 AEs. None of the studies reported on QoL. Two RCTs (N = 211) compared IDA with doxorubicin (DOX). Neither study assessed OS. One study showed that there was no evidence for difference in DFS (63 patients; HR 0.62, 95% CI 0.34 to 1.14, P = 0.12; low quality of evidence). The main meta-analysis for CR rate showed an improved CR rate with IDA (two studies, 187 patients; RR 1.28, 95% CI 1.03 to 1.59, P = 0.02; low quality of evidence). Neither study provided data for the risks of death on induction therapy and relapse. One trial showed that there was no evidence for difference in the risk of grade 3/4 cardiac toxicity (one study, 100 patients; RR 0.31, 95% CI 0.01 to 7.39, P = 0.47; very low quality of evidence). Neither study reported on QoL. Two RCTs (N = 1037) evaluated IDA versus zorubicin (ZRB). Neither study assessed OS. One trial showed that there was no evidence for difference in DFS (one study, 155 patients; HR 1.25, 95% CI 0.83 to 1.88, P = 0.29; low quality of evidence). The main meta-analyses for CR and death on induction therapy both showed that there was no evidence for difference (CR rate: two studies, 964 patients; RR 1.04, 95% CI 0.96 to 1.13, P = 0.31; low quality of evidence. risk of death on induction therapy: two studies, 964 patients; RR 0.75, 95% CI 0.50 to 1.13, P = 0.17; moderate quality of evidence). Neither study reported the risks of relapse and grade 3/4 cardiotoxicity. One trial showed that IDA reduced the risk of grade 3/4 mucositis. Neither study reported on QoL. AUTHORS’ CONCLUSIONS: Compared with DNR in induction therapy of newly diagnosed AML, IDA prolongs OS and DFS, increases CR rate and reduces relapse rate, although increases the risks of death on induction therapy and grade 3/4 mucositis. The currently available evidence does not show any difference between IDA and MIT used in induction therapy of newly diagnosed AML. There is insufficient evidence regarding IDA versus DOX and IDA versus ZRB to make final conclusions. Additionally, there is no evidence for difference on the effect of IDA compared with DNR, MIT, DOX or ZRB on QoL.) 10.1002/14651858.CD010432.pub2 PMC11218035 26037486

[21] Li XY, et al. [Analysis on clinical response of IA and DA regimens in the treatment of 74 newly diagnosed acute myeloid leukemia patients]. Zhonghua Xue Ye Xue Za Zhi 2013;34:67–68. 23597470

[22] Lowenberg B, et al. Acute myeloid leukemia. N Engl J Med 1999;341:1051–62. 10502596 10.1056/NEJM199909303411407

[23] Mandelli F, et al. Daunorubicin versus mitoxantrone versus idarubicin as induction and consolidation chemotherapy for adults with acute myeloid leukemia: the EORTC and GIMEMA Groups Study AML-10. J Clin Oncol. 2009;27(32):5397–403. (PURPOSE: To compare the antitumor efficacy of three different anthracyclines in combination with cytarabine and etoposide in adult patients with newly diagnosed acute myeloid leukemia (AML). PATIENTS AND METHODS: We randomly assigned 2,157 patients (age range, 15 to 60 years) to receive intensive induction-consolidation chemotherapy containing either daunorubicin, idarubicin, or mitoxantrone. After achieving complete remission (CR), patients were assigned to undergo either allogeneic or autologous stem-cell transplantation (SCT), depending on the availability of a sibling donor. RESULTS: The overall CR rate (69%) was similar in the three groups. Autologous SCT was performed in 37% of cases in the daunorubicin arm versus only 29% and 31% in mitoxantrone and idarubicin, respectively (P <.001). However, the disease-free survival (DFS) and survival from CR were significantly shorter in the daunorubicin arm: the 5-year DFS was 29% versus 37% and 37% in mitoxantrone and idarubicin, respectively. The proportion of patients who underwent allogeneic SCT (22%) was equivalent in the three treatment groups, and the outcome was similar as well. The [corrected] 5-year overall survival rates were 31%, 34%, and 34%, [corrected] respectively. CONCLUSION: In adult patients with AML who do not receive an allogeneic SCT, the use of mitoxantrone or idarubicin instead of daunorubicin enhances the long-term efficacy of chemotherapy.) 19826132 10.1200/JCO.2008.20.6490 PMC2773224

[24] Marschalek R. Mixed lineage leukemia: roles in human malignancies and potential therapy. Febs J. (2010). 277 (8):1822–31. (The increasing number of chromosomal rearrangements involving the human MLL gene, in combination with differences in clinical behavior and outcome for MLL-rearranged leukemia patients, makes it necessary to reflect on the cancer mechanism and to discuss potential therapeutic strategies. To date, 64 different translocations have been identified at the molecular level. With very few exceptions, most of the identified fusion partner genes encode proteins that display no homologies or functional equivalence. Only the most frequent fusion partners (AF4 family members, AF9, ENL, AF10 and ELL) are involved in the positive transcription elongation factor b-dependent activation cycle of RNA polymerase II. Biological functions remain to be elucidated for the other fusion partners. This minireview tries to sum up some of the available data and mechanisms identified in leukemic stem and leukemic tumor cells and link this information with the known functions of mixed lineage leukemia and certain mixed lineage leukemia fusion partners.) 20236311 10.1111/j.1742-4658.2010.07608.x

[25] Mehdipour P, et al. Epigenetic alterations in acute myeloid leukemias. Febs J. 2015;282(9):1786–800. (Acute myeloid leukemia (AML) is a heterogeneous disease for which the standard treatment with cytotoxic chemotherapy has remained largely unchanged for over four decades, with unfavorable clinical results. Epigenetic alterations have been described in several AMLs, and in some cases their origin has been studied in detail mechanistically (such as in acute promyelocytic leukemia, caused by the promyelocytic leukemia-retinoic acid receptor-α fusion protein). Recently, the advent of massive parallel sequencing has revealed that > 70% of AML cases have mutations in DNA methylation-related genes or mutations in histone modifiers, showing that epigenetic alterations are key players in the development of most, if not all, AMLs, and pointing to the exploitation of new molecular targets for more efficacious therapies. This review provides a brief overview of the latest findings on the characterization of the epigenetic landscape of AML and discusses the rationale for the optimization of epigenetic therapy of AML.) 25369368 10.1111/febs.13142

[26] Network NCC. NCCN clinical practice guidelines in oncology Acute Myeloid Leukemia (version 2.2016). 2016 Available from: https://www.nccn.org/professionals/physician_gls/pdf/aml.pdf. Accessed 29 June 2016

[27] Network NCC. NCCN clinical practice guidelines in oncology Hematopoietic Cell Transplantation (version 5.2021). 2021.Available from: https://www.nccn.org/professionals/physician_gls/pdf/hct.pdf. Accessed 30 Sep 2021

[28] Ohtake S, et al. Randomized study of induction therapy comparing standard-dose idarubicin with high-dose daunorubicin in adult patients with previously untreated acute myeloid leukemia: the JALSG AML201 Study. Blood. 2011;117(8):2358–65. (We conducted a multi-institutional randomized study to determine whether high-dose daunorubicin would be as effective as standard-dose idarubicin in remission-induction therapy for newly diagnosed adult patients younger than 65 years of age with acute myeloid leukemia. Of 1064 patients registered, 1057 were evaluable. They were randomly assigned to receive either daunorubicin (50 mg/m(2) daily for 5 days) or idarubicin (12 mg/m(2) daily for 3 days) in combination with 100 mg/m(2) of cytarabine by continuous infusion daily for 7 days as induction therapy. Complete remission was achieved in 407 (77.5%) of 525 patients in the daunorubicin group and 416 (78.2%) of 532 in the idarubicin group (P =.79). Patients achieving complete remission received intensive postremission therapy that consisted of either 3 courses of high-dose cytarabine or 4 courses of standard-dose therapy. Overall survival rates at 5 years were 48% for the daunorubicin group and 48% for the idarubicin group (P =.54), and relapse-free survival rates at 5 years were 41% and 41% (P =.97), respectively. Thus, high-dose daunorubicin and standard-dose idarubicin were equally effective for the treatment of adult acute myeloid leukemia, achieving a high rate of complete remission and good long-term efficacy. This study is registered at http://www.umin.ac.jp/ctrj/ as C000000157.) 20693429 10.1182/blood-2010-03-273243

[29] Ren X, et al. [Factors associated with early treatment response in adults with acute myeloid leukemia]. Zhonghua Xue Ye Xue Za Zhi. 2017;38(10):869–75. (Objective: To explore the factors influencing early treatment responses in adult with de novo acute myeloid leukemia (AML). Methods: Data of consecutive newly-diagnosed AML (non-acute promyelocytic leukemia) adults were analyzed retrospectively. To assess the impact of clinical characteristics at diagnosis and induction regimen on achieving morphologic leukemia-free state (MLFS), blood counts and minimal residual leukemia (MRD, positive MRD defined as RQ-PCR WT1 mRNA >/=0.6% and/or any level of abnormal blast population detected by flow cytometry) at the time of achieving MLFS. Results: 739 patients were included in this study. 406 (54.9%) patients were male, with a median age of 42 years (range, 18–65 years). In the 721 evaluable patients, MLFS was achieved in 477 (66.2%) patients after the first induction regimen and 592 (82.1%) within two cycles. A total of 634 patients (87.9%) achieved MLFS, including 534 (84.2%) achieving a complete remission (CR, defined as MLFS with ANC >/= 1×10(9)/L and PLT >/= 100×10(9)/L), 100 (15.8%) achieving a CRi (defined as MLFS with incomplete ANC or PLT recovery), respectively. 260 (45.9%) patients of 566 (89.3%) who detected MRD at the time of achieving MLFS had positive MRD. Multivariate analyses showed that female gender, favorable-risk of SWOG criteria, IA10 and HAA/HAD as induction regimen were factors associated with achieving early MLFS. In addition, low bone marrow blasts, HGB >/= 80 g/L, PLT counts<30×10(9)/L and mutated NPM1 without FLT3-ITD were factors associated with achieving MLFS after the first induction regimen; Negative FLT3-ITD mutation was factor associated with achieving MLFS within two cycles. PLT counts >/=30×10(9)/L and IA10, IA8 or HAA/HAD as induction chemotherapy were factors associated with achieving CR. Female gender, favorable-risk of SWOG criteria, FLT3-ITD mutation negative, mutated NPM1 without FLT3-ITD were factors associated with negative MRD. Conclusions: Female gender, favorable molecular markers or cytogenetics, and standard-dose induction regimen were key factors associated with higher probability of early and deep responses in adults with AML.) 29166740 10.3760/cma.j.issn.0253-2727.2017.10.009 PMC7364970

[30] Ren X, et al. [Outcomes of adult patients with de novo acute myeloid leukemia received idarubicin plus cytarabine regimen as induction chemotherapy]. Zhonghua Xue Ye Xue Za Zhi. 2018;39(1):15–21. (Objective: To explore outcomes in adult with de novo acute myeloid leukemia (AML) received IA10 (10 mg/m(2) d1-3 idarubicin plus cytarabine 100 mg/m(2) d1-7) regimen as induction chemotherapy. Methods: From January 2008 to February 2016, data of consecutive newly-diagnosed AML (non-M(3)) adults treated with IA10 who achieved morphologic leukemia-free state (MLFS) but not accepted allogeneic hematopoietic stem cell transplantation (allo-HSCT) were assessed retrospectively. Results: A total of 198 patients were included in this study with 96 (48.5%) male and a median age of 42 years old (range, 18–62 years old). Using the SWOG cytogenetic classification, 45 (22.7%), 104 (52.5%), 24 (12.1%) and 25 (12.6%) patients belonged to favorable, intermediate, unfavorable and unknown categories, respectively. 6 (3.0%) patients had monosomal karyotype, and 28 (14.1%) positive FLT3-ITD mutation. A complete remission (CR, defined as MLFS with ANC >/= 1×10(9)/L and PLT >/= 100×10(9)/L) achieved in 168 (84.8%) patients, a CRp (defined as MLFS with incomplete PLT recovery) in 16 (8.1%) and a CRi (defined as MLFS with incomplete ANC and PLT recovery) in 14 (7.1%). With a median follow-up period of 15 months (range, 1 to 70 months) in survivors, the probabilities of cumulative incident of relapse (CIR), disease free survival (DFS) and overall survival (OS) rates at 2-year were 45.2%, 46.9% and 62.9%, respectively; the median durations of relapse, DFS and OS were 34, 20 and 37 months respectively. At the time of achieving first MLFS, multivariate analyses showed that positive FLT3-ITD mutation and CRi were common adverse factors affecting CIR, DFS and OS; unfavorable-risk of SWOG criteria was an adverse factor affecting CIR and DFS; monosomal karyotype was associated with shorter OS. After first consolidation therapy, FLT3-ITD mutation positive and unfavorable-risk of SWOG criteria had negatively impact on CIR, DFS and OS; peripheral blasts >/= 0.50 and positive MRD (defined as RQ-PCR WT1 mRNA >/= 0.6% or any level of abnormal blast population detected by flow cytometry) after first consolidation therapy were common adverse factors affecting CIR and DFS; CRi was an adverse factor affecting DFS and OS. Conclusions: In adult with de novo AML received IA10 regimen as induction regimen, unfavorable molecular markers or cytogenetics at diagnosis and CRi independently predicted poor outcome. In addition, a higher percentage of peripheral blasts, monosomal karyotype and positive MRD after first consolidation therapy had negatively impact on outcomes.) 29551027 10.3760/cma.j.issn.0253-2727.2018.01.004 PMC7343116

[31] Robert J, et al. Comparative pharmacokinetic study of idarubicin and daunorubicin in leukemia patients. Hematol Oncol. 1992;10(2):111–16. (We have studied the pharmacokinetics of idarubicin and daunorubicin in a total of 16 leukemic patients treated with one of these drugs associated with aracytine. The AUCs obtained for unchanged drugs were proportional to the dose, and the dose-independent pharmacokinetic parameters were very similar for the two drugs: total plasma clearance (39.0 L/h/m2 for idarubicin versus 38.6 for daunorubicin), total volume of distribution (1756 versus 1725 L/m2) and elimination half-life (42.7 versus 47.4 h). The only metabolites detected were the 13-dihydroderivative of each drug, idarubicinol or daunorubicinol. The elimination half-life of idarubicinol was two times higher than that of daunorubicinol (80.7 versus 37.3 h) which provided an AUC ratio metabolite/parent drug higher for idarubicin than for daunorubicin. In view of the fact that idarubicinol is a much more active metabolite than daunorubicinol, this protracted half-life metabolite can account for the reported higher activity of idarubicin as compared to daunorubicin.) 1592361 10.1002/hon.2900100207

[32] Ruggeri A, et al. Bone marrow versus mobilized peripheral blood stem cells in haploidentical transplants using posttransplantation cyclophosphamide. Cancer. 2018;124(7):1428–37. (BACKGROUND: Incidence of graft-versus-host disease (GVHD) in haploidentical bone marrow (BM) transplants using posttransplantion cyclophosphamide (PT-Cy) is low, whereas GVHD using mobilized peripheral blood stem cells (PBSC) ranges between 30% and 40%. METHODS: To evaluate the effect of stem cell source in haploidentical transplantation with PT-Cy, we analyzed 451 patients transplanted for acute myeloid leukemia or acute lymphoblastic leukemia reported to the European Society for Blood and Marrow Transplantation. RESULTS: BM was used in 260 patients, and PBSC were used in 191 patients. The median follow-up was 21 months. Engraftment was lower in BM (92% vs 95%, P < 0.001). BM was associated with a lower incidence of stage II-IV and stage III-IV acute GVHD (21% vs 38%, P </=.01; and 4% vs 14%, P <.01, respectively). No difference in chronic GVHD, relapse, or nonrelapse mortality were found for PBSC or BM. The 2-year overall survival (OS) was 55% versus 56% (P =.57) and leukemia-free survival (LFS) was 49% versus 54% (P =.74) for BM and PBSC, respectively. On multivariate analysis, PBSC were associated with an increased risk of stage II-IV (hazard ratio [HR], 2.1; P <.001) and stage III-IV acute GVHD (HR, 3.8; P <.001). For LFS and OS, reduced intensity conditioning was the only factor associated with treatment failure (LFS: HR, 1.40; P =.04) and relapse (HR, 1.62; P =.02). CONCLUSION: In patients with acute leukemia in first or second remission receiving haploidentical transplantation with PT-Cy, the use of PBSC increases the risk of acute GVHD, whereas survival outcomes are comparable. Cancer 2018;124:1428-37. (c) 2018 American Cancer Society.) 29360162 10.1002/cncr.31228

[33] Sharvit G, et al. Acute myeloid leukemia patients requiring two cycles of intensive induction for attainment of remission experience inferior survival compared with patients requiring a single course of induction chemotherapy. Clin Lymphoma Myeloma Leuk. 2022;22(2):e116–e123. (BACKGROUND: Achievement of initial remission remains the most important clinical factor predicting long term survival in acute myeloid leukemia (AML) patients treated with intensive chemotherapy. Yet, whether the patient subset in need of a second cycle of intensive induction chemotherapy to reach remission experiences inferior outcomes compared to patients reaching remission after a single cycle of therapy, remains uncertain. PATIENTS AND METHODS: Retrospective analysis of 302 consecutive AML patients treated with intensive induction chemotherapy in our institution in 2007-2020. RESULTS: Median patient age was 55 years with a median follow-up duration of 23 months. In terms of European LeukemiaNet (ELN) 2017 classification, 122 patients (40%) were designated as favorable risk disease, 108 patients (36%) were intermediate risk, and 71 patients (24%) were adverse risk. A hundred and seventy-seven patients (60%) attained remission following initial chemotherapy while 58 patients (20%) required an additional cycle of intensive chemotherapy for remission. Patients requiring 2 cycles to reach remission were less likely to be NPM1 mutated (33% versus 51%; P=.025) or be in the ELN 2017 favorable risk category (25% versus 57%; P<.001). In multivariate analysis achievement of remission following 2 cycles of intensive compared with a single cycle resulted in significantly inferior survival [hazard ratio (HR)=1.67, 95% CI, 1.07-2.59; P=.025] whereas leukemia-free survival was not significantly impacted (HR=1.26, 95% CI, 0.85-1.85) (P=.23). Relapse rates also did not differ to a significant degree between groups (45% versus 47%, P=.8). CONCLUSION: Attainment of an early remission significantly impacts long term survival in AML patients.) 34593360 10.1016/j.clml.2021.08.014

[34] Shlush LI, et al. Identification of pre-leukaemic haematopoietic stem cells in acute leukaemia. Nature. 2014;506(7488):328–33. (In acute myeloid leukaemia (AML), the cell of origin, nature and biological consequences of initiating lesions, and order of subsequent mutations remain poorly understood, as AML is typically diagnosed without observation of a pre-leukaemic phase. Here, highly purified haematopoietic stem cells (HSCs), progenitor and mature cell fractions from the blood of AML patients were found to contain recurrent DNMT3A mutations (DNMT3A(mut)) at high allele frequency, but without coincident NPM1 mutations (NPM1c) present in AML blasts. DNMT3A(mut)-bearing HSCs showed a multilineage repopulation advantage over non-mutated HSCs in xenografts, establishing their identity as pre-leukaemic HSCs. Pre-leukaemic HSCs were found in remission samples, indicating that they survive chemotherapy. Therefore DNMT3A(mut) arises early in AML evolution, probably in HSCs, leading to a clonally expanded pool of pre-leukaemic HSCs from which AML evolves. Our findings provide a paradigm for the detection and treatment of pre-leukaemic clones before the acquisition of additional genetic lesions engenders greater therapeutic resistance.) 24522528 10.1038/nature13038 PMC4991939

[35] Wang H, et al. The efficacy and safety of daunorubicin versus idarubicin combined with cytarabine for induction therapy in acute myeloid leukemia: a meta-analysis of randomized clinical trials. Medicine. 2020;99(24):e20094. (Objective: To ascertain the efficacy and safety of daunorubicin combined with cytarabine comparing with idarubicin combined with cytarabine as a standard induction therapy for acute Myeloid leukemia by a meta-analysis. Methods: The randomized controlled trials included were retrieved from PubMed, Embase, and Cochrane library. We evaluated and cross-checked the randomized clinical trials (RCTs) comparing daunorubicin combined with cytarabine (DA) and idarubicin combined with cytarabine (IA) by two reviewers independently according to Cochrane Handbook for Systematic Reviewers of Interventions. The data of meta-analysis was conducted using Review Manager 5.3 and Stata 12.0 software. Results: A total of 6 studies containing 3140 patients were included. The primary outcomes were complete remission (CR), CR in one course (CR1), CR in two courses (CR2), overall survival (OS), and relapse rate. The secondary outcomes included adverse events and cytogenetic risk in subgroup analyses. IA showed a statistically significant in CR (RR = 1.05; 95%CI = 1.00–1.09, P =.03) and CR1 (RR = 1.11; 95%CI = 1.04–1.18, P =.003), but not in CR2 (RR = 0.97; 95%CI = 0.77–1.24, P =.83), and relapse rate (RR = 1.08; 95%CI = 0.98–1.43, P =.08). In high dose daunorubicin group, OS was significantly improved with IA compared to DA (HR = 0.89, 95%CI = 0.8–1.0, P =.041, I2 = 0). At grade 3/4 adverse events, the difference between IA and DA was not statistically significant (infection, P =.28; cardiac toxicity, P =.15; bleeding, P =.29). In the subgroup analysis, the genotypes of the IA and DA groups were not statistically significant for comparison of CR between the two groups (P =.07). Conclusion: This meta-analysis showed that IA had a better efficacy in the treatment of acute myeloid leukemia than DA, even with increased doses of DA. The OS of a standard dose of IA patients was longer than that of DA patients. Our research shows that anthracycline dose intensification of daunorubicin is of no clinically relevant benefit in AML patients comparing with a standard dose of IA. When it comes to adverse drug reactions, it is not a significant difference. Therefore, in clinical practice, IA should be the first choice for induction regimen in patients with acute myeloid leukemia.) 32541448 10.1097/MD.0000000000020094 PMC7302600

[36] Wiernik PH, et al. Cytarabine plus idarubicin or daunorubicin as induction and consolidation therapy for previously untreated adult patients with acute myeloid leukemia. Blood. 1992;79(2):313–19. (The purpose of this study was to determine the relative merits of idarubicin and daunorubicin in acute myeloid leukemia (AML) therapy. Thirty-two sites provided 214 previously untreated adults with AML aged 15 years or more who were randomized to receive for induction therapy cytarabine 100 mg/m2/d as a continuous 7-day infusion plus either daunorubicin 45 mg/m2/d (A + D) or idarubicin 13 mg/m2/d (A + I), daily on the first three days of treatment. Postremission therapy consisted of two courses of the induction regimen at the same daily doses, with the anthracycline administered for 2 days and cytarabine for 5. The complete response (CR) rates for evaluable patients were 70% (A + I) and 59% (A + D) (P =.08). The difference in CR rates was significant in patients aged 18 to 50 years (88% for A + I, 70% for A + D, P =.035). Resistant disease was a significantly more frequent cause of induction therapy failure with A + D than with A + I. Hyperleukocytosis (white blood cell count greater than 50,000/microL) unfavorably affected the attainment of CR with A + D but not with A + I. CR duration was significantly greater after A + I. CR duration was significantly greater after A + I treatment, and the survival of all randomized patients treated with A + I was significantly better than that observed after A + D treatment (median 12.9 months v 8.7 months, respectively, P =.038). Toxicity of the two treatments was similar, although A + I patients experienced more prolonged myelosuppression during consolidation therapy, and a greater incidence of mild chemical hepatitis was observed in the A + I group. It is concluded that, at the doses and schedule used in this study, A + I is superior to A + D for induction therapy of AML in adults.) 1730080

